# Exploring changes in levels and patterns of physical activity in undergraduate medical and nursing students during the COVID-19 pandemic

**DOI:** 10.3389/fpubh.2022.1042071

**Published:** 2022-10-26

**Authors:** Ahmed Al-Hindawi, Nitya Kumar, Declan Gaynor

**Affiliations:** School of Medicine, Royal College of Surgeons in Ireland Medical University of Bahrain, Muharraq, Bahrain

**Keywords:** COVID-19, social distancing, physical activity, sedentary behavior, university student

## Abstract

This study reports the physical activity (PA) levels among medical and nursing students at a university in Bahrain during the COVID-19 pandemic. Through self-selection sampling of an online survey, participants' data on general demographics, PA levels before and during the COVID-19 pandemic and reasons for PA changes were collected. From the 110 valid responses, 70 participants (63%) experienced a decrease in PA during the COVID-19 lockdown. Fear of contracting COVID-19 and lack of motivation were two significant reasons for reduced PA levels (*p* < 0.001) compared to those who did not experience a decrease in PA. Other factors significantly associated with reduced PA levels include living alone (*p* < 0.018) or with roommates (*p* < 0.006) compared to living with family. Having more time available was associated with positive changes to PA levels (*p* < 0.001). Significant differences in MET-min/week were seen between students who experienced increased PA (median of 1605 MET-min/week) compared to those who experienced a decrease (424 MET-min/week) or no change (1070 MET-min/week) in PA levels (*p* < 0.001). In conclusion, low PA levels are prevalent within medical and nursing students in Bahrain (51% reported < 600 MET-min/week), with ~2 in 3 students reporting a decrease in PA levels during the COVID-19 pandemic. Support programs and strategies to increase engagement in PA within this population are warranted.

## Introduction

In December 2019, a new form of coronavirus (SARS-CoV-2) was identified in China (1). Over the course of a few months, coronavirus disease 2019 (commonly known as COVID-19) caused by SARS-CoV-2 has been declared a pandemic and a global public health emergency by the World Health Organization on 11 March 2020 (2).

As of 16 August 2022, COVID-19 has infected more than 593 million people and led to at least 6.4 million deaths globally (3). In the majority of the cases, COVID-19 is described as an acute respiratory illness with its common symptoms being fever, cough, and a sore throat. However, in moderate-to-severe cases, the disease progresses to cause difficulties in breathing, complications in non-respiratory organs including the heart and kidneys, and death (4).

As a means to reduce the risk of contracting the virus and curbing its spread, countries around the world have adopted community and nation-wide lockdowns (5). In addition, other preventive measures include home quarantines, self-isolation, working-from-home, social distancing, and the prohibition of social gatherings. For most university students, this also entailed the closure of campuses and facilities, transitioning to remote/online learning, and returning to their home countries in the case of international students.

The wildfire-like spread of COVID-19 and the drastic measures governments and public health agencies have taken has brought about a plethora of new challenges, one of which is the profound disruption of the daily routines and lifestyles of the general population (6). While effective for limiting the spread of outbreaks, the strict isolation imposed by governments can negatively impact physical and mental health; this could be further exacerbated among young adults who rely on cues such as positive peer interactions and social gatherings for their general well-being (7, 8).

Due to the aforementioned restrictions, it is reasonable to assume that university students' physical activity and exercise behaviors have been altered over the course of the lockdown period. In fact, inadequate physical activity levels were already described as a global health problem before lockdown, with over a quarter of all adults (9) and three-fourth of adolescents (10) not meeting the minimum recommendations for physical activity [150 min of moderate-intensity physical activity, or 75 min of vigorous-intensity physical activity, or an equivalent combination of the two, per week (11)]. Further, sedentary behavior within university students has been gradually increasing over the past 10 years, with current estimates exceeding 9 h of sedentary time per day (12).

The importance of physical activity should be highlighted as it is linked to the prevention of non-transmissible chronic diseases (such as cardiovascular disease, hypertension, and diabetes) (13) and improved mental health outcomes (14). Indeed, regular physical activity is deemed to be an efficacious strategy to prevent premature death (15, 16). Consequently, academics are continuing to encourage people to exercise during these unprecedented times (17, 18).

Students' inactivity may have worsened due to the inaccessibility of gyms and home quarantine requirements, which have limited options for exercise. On the other hand, the less rigid scheduling and the arguable increase in disposable time may encourage students to exercise more frequently. Recent studies have explored the potential fluctuations of physical activity within the general population during lockdown. Numerous studies carried out in China have reported a considerable decrease in physical activity within the general population (19–21). Furthermore, a few studies conducted in Europe have also reported similar results (22–24). Yet, other studies have reported conflicting results where there was an increase in physical activity during lockdown (25, 26). From this, one can see that the rather limited conclusions on this topic are varied and inconclusive.

Empirical evidence concerning the effects of lockdown on university students specifically is limited, a population already at risk of low physical activity. A study conducted within an Australian university reported a reduction in physical activity within its students when compared to previous years (27). Two studies of undergraduate university students in South America also reported decreased physical activity during compared to before the COVID-19 pandemic (28, 29). Likewise, a study involving physiotherapy students reported a significant reduction in self-reported physical activity and energy expenditure levels (30). On the other hand, health science students within a Spanish university saw an increase in both weekly physical activity and sitting time (31). However, in the context of medical and nursing university students, particularly within the Middle East and North African (MENA) region, the topic has been largely unexplored.

This aim of this study was to examine any changes to physical activity levels and patterns as a result of the enforced lockdown within medical and nursing university students when compared to pre-lockdown levels. We also intended to report on any likely variables that may have influenced potential changes. This study may provide valuable information on an unreported population to assess the severity of the lockdown's effect on physical activity and determine if there is a need to develop action plans or policies to encourage maintenance of physical activity levels in students during extended periods of lockdown and social distancing measures.

## Materials and methods

### Study design, setting, and sample size

A cross-sectional study design was implemented in this research study which was undertaken at an international university in the Kingdom of Bahrain. The university has ~1,300 enrolled full-time students. During the first semester of the 2020/2021 academic year, all accessible full-time undergraduate students who were 18 years or older and enrolled students at the university during the previous academic year (2019/2020) were invited to join the study. Participants completed an anonymous online survey which contained the International Physical Activity Questionnaire (IPAQ) and additional items related to the participants' demographics and both their levels and patterns of physical activity during the COVID pandemic. Results were analyzed to determine any self-reported changes in physical activity behavior and the factors which may be associated with these changes.

To estimate required sample size for determining the proportion of individuals who would have experienced a decrease in levels of physical activity, we considered the reported proportion in literature of 21% (25) confidence level of 95%, a 5% margin of error, a finite population size of 1,000 individuals and obtained the size to be 204 individuals, using the approach described by Daniel and Cross (32). To ensure the multivariate logistic regression models would not be overfit, we considered minimum of 15 outcome events (i.e., individuals who experienced decrease in physical activity during pandemic) for each covariate we intend to include in the model (33), which is four. Given the reported proportion of outcome is 21%, the minimum sample to run the logistic regression model was found to be 287. Considering both the above approaches, the proposed sample size for this study was estimated to be 290.

### Recruitment of participants and data collection

Self-selection sampling was used to recruit the participants in the study. Approximately 1,000 students of the 1,300 enrolled students were invited to participate in the study *via* email through the University network. The email provided participants with the participant information leaflet and a link to the online questionnaire which was delivered *via* the university licensed Microsoft Office Forms service in anonymous mode so that no unique identifier was collected from any participants. Before completing the survey participants provided informed consent *via* the online form. Participants were provided with an opportunity to withdraw from the study at any time even after submission of their survey responses through the use of a “memorable phrase” item within the questionnaire.

In addition to the items contained within the IPAQ, the questionnaire included items on basic demographics such as gender, programme of study (medicine or nursing), year of study and living situation (living at home with family, living alone, living with other students). The questionnaire also consisted of items based on a recently published large population study in Belgium (25). These items measured (a) physical activity level in the months immediately before the pandemic; (b) self-reported pattern of physical activity before the pandemic; (c) level of physical activity and sedentary behavior during the pandemic compared to before it; (d) factors which positively and negatively contributed to changes in physical activity during the pandemic; and (e) usage of a smart watch or online physical activity or exercise tracking app. Items measuring physical activity levels before and during the pandemic and contributing factors are outlined in Table 1.

**Table 1:** Items measuring physical activity levels, patterns and contributing factors before and during period of COVID-19 pandemic. Adapted from Constandt et al. (25).

**Item**	**Response options**
(1) Select the response that best describes your level of physical activity in the months before the COVID-19 pandemic.	(a) I exercised regularly at least once a week. (b) I did not exercise at all or less than once a week
(2) Select the most relevant option(s) for completing the statement below. Before the pandemic I was mainly exercising ________.	(a) alone (b) with friends (c) with family (d) with online support (e.g., Strava, MapMyRun, Fitbit) (e) at a sports facility or gym
(3) During the period from March to October 2020, my physical activity and exercise levels have decreased compared to before the pandemic started.	(a) Yes (b) No
(4) During the period from March to October 2020 my physical activity and exercise levels have increased compared to before the pandemic.	(a) Yes (b) No
(5) During the period from March to October 2020 my sedentary behavior (time spent sitting) has decreased compared to before the pandemic.	(a) Yes (b) No
(6) During the period from March to October 2020 my sedentary behavior (time spent sitting) has increased compared to before the pandemic.	(a) Yes (b) No
(7) Select the most relevant option(s) for completing the statement below. During the period from March to October 2020 my physical activity and exercise habits have been negatively impacted by __________.	(a) having fear of contracting COVID-19 (b) closed sports facilities and gyms (c) canceled sports events (d) having no or reduced motivation (e) having less or little available time (f) being ill (g) nothing
(8) Select the most relevant option(s) for completing the statement below. During the period from March to October 2020 my physical activity and exercise habits have been positively impacted by ___________.	(a) providing support to family member (b) having more available time (c) having more motivation (d) nothing
(9) I use a smart watch and/or activity tracking apps on my phone to track my physical activity and exercise	(a) Yes (b) No

### Data analysis

The study design required participants to be age 18 year or more and be a registered student at the Royal College of Surgeons in Ireland Medical University of Bahrain during the academic year of 2019/2020. Of the 126 responses recorded, a total of 110 were included in the analysis after excluding all incomplete questionnaires. Data from all valid participants' questionnaire responses were compiled and analyzed using Microsoft Excel (RRID:SCR_016137) and STATA 13 (RRID:SCR_012763). The IPAQ data was cleaned, processed and Metabolic Equivalent Task minutes/week (MET-minutes/week) were calculated according to the IPAQ scoring protocol document (34). Of the 110 participants included in the survey analysis 105 of these provided valid responses to the IPAQ instrument according to the IPAQ protocols. Changes in the level of physical activity during the pandemic are reported using descriptive statistics. To study the association between change in physical activity and the measured covariates, participants were first categorized in three separate groups according to self-reported changes in their levels of physical activity (PA) immediately before and during the COVID-19 pandemic, (1) No change in PA (2) Decreased PA (3) Increased PA. To look at the determinants of decrease in PA, multivariate logistic regression models were built with decreased PA as an outcome (compared to no change or increase in PA). Similarly, to study determinants of increase in PA multivariate regression models with increase in PA as the outcome (compared to no change or decrease in PA) were fit. Models selection was based on parameters of goodness of fit, calibration and discrimination.

## Results

It was only possible to contact a section of the eligible student population and keep the questionnaire link accessible for a limited period due to institution-imposed limitations. As a result, the total number of valid questionnaire responses (110) did not meet the sample size calculation of 290 based for the proposed logistic regression analysis. Despite not meeting the sample size requirements, the proposed logistic regression analysis was completed and returned goodness of fit metrics which indicated good calibration with Pearson χ2 when examining factors which were associated with both a decrease and increase in physical activity during the pandemic.

### Descriptive statistics

Of the 110 valid participant's responses, 68 (62%) were female and 42 (38%) were male (Table 2). 98 (89%) were medical students and 12 (11%) were nursing students. 56 (51%) reported living at home with family while the remainder either lived alone or with room-mates. 70 (63%) participants reported experiencing a decrease in their level of physical activity during the COVID-19 social distancing measures compared to the 3 months pre-COVID-19 (Table 2). An increase in physical activity was reported by 30 (27%) of the participants and 10 (9%) participants indicated they experienced no change in their levels of physical activity.

**Table 2:** Participant characteristics according to change in physical activity (PA) levels during COVID-19 social distancing measures^#^.

**Factor**	**Total**	**No change in PA**	**Decreased PA**	**Increased PA**	***p*-value**
*N*	110 (100%)	10 (9%)	70 (64%)	30 (27%)	
**Gender**					
Female	68 (62%)	4 (40%)	43 (61%)	21 (70%)	0.24
Male	42 (38%)	6 (60%)	27 (39%)	9 (30%)	
**Programme of study**					
Nursing	12 (11%)	1 (10%)	7 (9.7%)	4 (13%)	0.86
Medicine	98 (89%)	9 (90%)	65 (90.3%)	26 (86.7%)	
**Living situation**					
Home with family	56 (51%)	7 (70%)	30 (43%)	19 (63%)	0.25
Alone	41 (37%)	2 (20%)	30 (43%)	9 (30%)	
With room-mates	13 (12%)	1 (10%)	10 (14%)	2 (7%)	
**Sedentary behavior**					
No change in sedentary behavior during COVID-19	6 (6%)	2 (22%)	1 (1%)	3 (10%)	< 0.001
Decreased sedentary behavior during COVID-19	29 (26%)	1 (11%)	14 (20%)	14 (47%)	
Increased sedentary behavior during COVID-19	75 (68%)	6 (67%)	56 (79%)	13 (43%)	
**Physical activity behavior**					
Physically active before COVID-19	63 (57%)	6 (60%)	38 (54%)	19 (63%)	0.69
Used to work out alone pre-covid	58 (53%)	5 (50%)	34 (49%)	19 (63%)	0.43
Used to work out with Friends pre-covid	38 (35%)	2 (20%)	26 (38%)	10 (33%)	0.54
Used to work out with family pre-covid	7 (6%)	0 (0%)	6 (9%)	1 (3%)	0.42
Used to work out with online support pre-covid	9 (8%)	2 (20%)	3 (4%)	4 (13%)	0.12
Used to work out in a sports facility or gym pre-covid	41 (37%)	4 (40%)	32 (46%)	5 (17%)	0.019
Use smart watch	47 (43%)	5 (50%)	30 (43%)	12 (41%)	0.89

# Values in parentheses are column percentages. All p-values are from Chi-square test.

### Group comparison analysis

#### Group characteristics impact on changes in physical activity

According to Chi-square test results, gender, programme of study (Medicine, Nursing) and living situation (living at home with family, living alone, living with roommates) had no significant association with the reported change in physical activity levels experienced by the participants.

Participants also reported the type of physical activity behaviors they engaged in before COVID-19, whether it was alone, with friends, with family, with online support or at a sports facility or gym. Table 2 shows the proportion of participants of each group (No Change in PA, Decreased PA, Increased PA) who reported the types of physical activity behaviors they engaged in prior to COVID-19.

Group differences were observed for reported changes in sedentary behavior during COVID-19, with 79% of Decreased PA group reporting increased sedentary behavior compared to 67% for No Change in PA group and 43% for Increased PA group. While 47% of Increased PA group reported decreased sedentary behavior compared to 11% for No change in PA group and 20% in Decreased PA group (*p* < 0.001) (Table 2).

**Figure 1:**
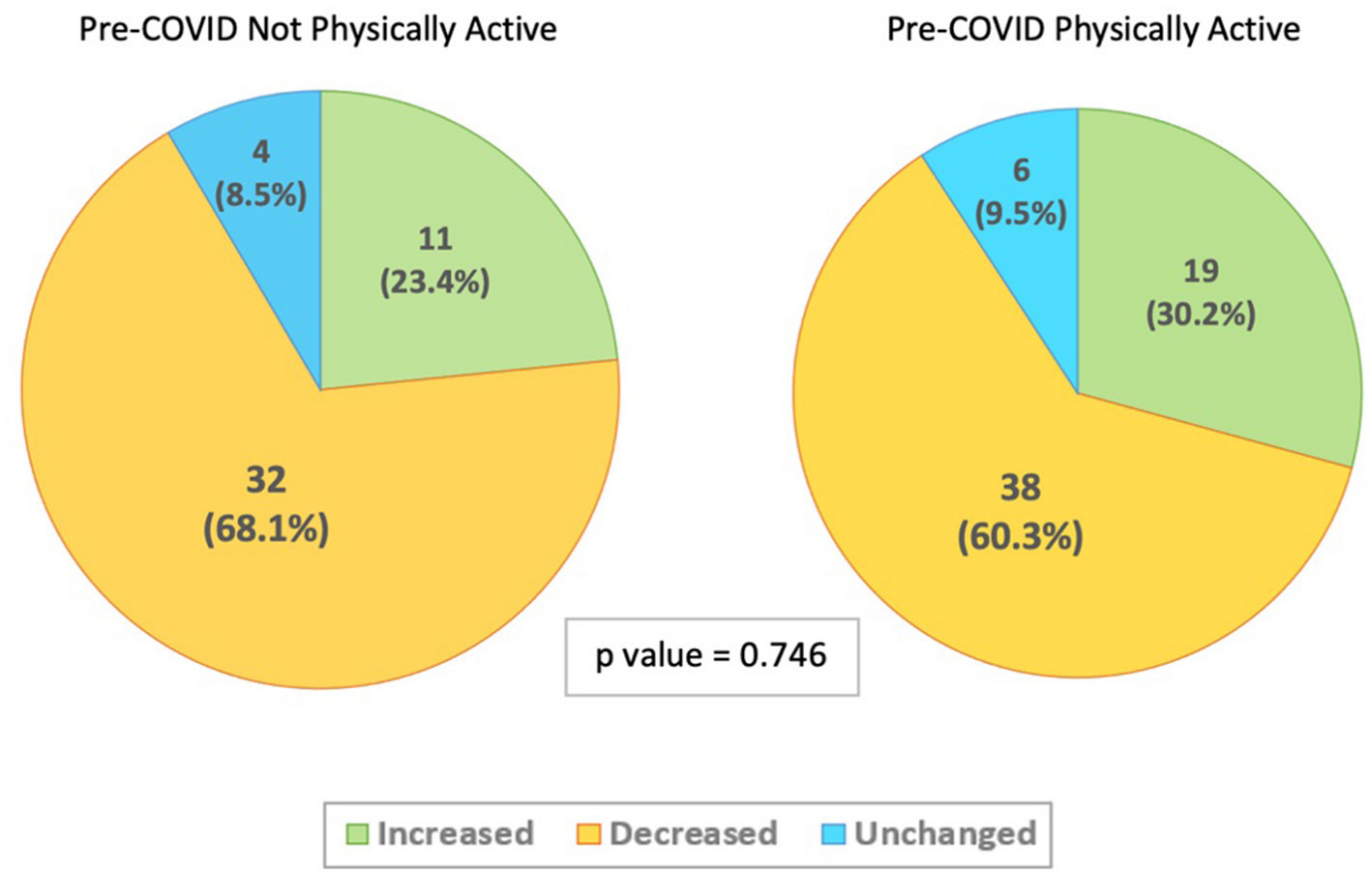
Change in physical activity levels during COVID-19 social distancing measures stratified by Pre-COVID-19 physical activity levels (*N* = 110).

There were no significant differences between the groups in relation to whether they were physically active pre-COVID-19 (Figure 1), exercised with a smart watch, exercised alone, exercised with others or used online support. However, there was a significant difference between the groups who reported engaging in physical activity at a sports facility or gym before COVID-19 (*p* = 0.019). Participants who reported increased physical activity during COVID-19 were much less likely to have engaged in physical activity at a sports center or gym (17%) pre-COVID-19 compared to those who experienced a decrease (46%) or no change in physical activity (40%).

#### Physical activity levels of groups during COVID-19 social distancing measures

The results of participant's IPAQ responses showed significant differences in the overall total number of MET-minutes/week between groups who indicated they experienced an increase, a decrease, or no change in the level of physical activity during COVID-19 (*p* = 0.001) (Table 3). The Increased PA group had a median of 1605 MET-minutes/week compared to 424 MET-minutes/week for Decreased PA group and 1070 MET-minutes/week for No Change in PA group. Similar trends in differences between the groups were observed for both the vigorous (*p* < 0.001) and moderate (*p* = 0.030) categories of PA but not in the walking category of PA.

**Table 3:** IPAQ physical activity data during COVID-19 social distancing measures according to change in physical activity (PA) levels^#^.

**Factor**	**Total**	**No change in PA**	**Decreased PA**	**Increased PA**	***p*-value**
N	105	10	68	27	
**IPAQ physical activity MET-minutes/week during COVID-19**					
Walking MET, median (IQR)		363 (132, 1,039)	148 (0, 495)	247 (66, 924)	0.23
Moderate MET, median (IQR)		0 (0, 160)	0 (0, 120)	100 (0, 420)	0.030
Vigorous MET, median (IQR)		280 (0, 160)	0 (0, 420)	960 (0, 1,920)	< 0.001
Total MET, median (IQR)		1,070 (316, 2,639)	424 (132, 1,399)	1,605 (852, 3,342)	0.001
**IPAQ physical activity level category during COVID-19**					
Low (< 600 MET minutes/week)	54 (51%)	4 (40%)	42 (62%)	8 (30%)	0.007
Moderate (≥600 and < 3,000 MET minutes/week)	28 (27%)	3 (30%)	18 (26%)	7 (26%)	
High (≥3,000 MET minutes/week)	23 (22%)	3 (30%)	8 (12%)	12 (44%)	

# Values in parentheses are column percentages for categorical variables and inter-quartile range for continuous variables. Significance values for Duration of PA during COVID-19 MET-minutes/week are from Kruskal-Wallis test. All other p-values are from Chi-square test.

#### Perceived factors affecting physical activity levels in groups during COVID-19 social distancing measures

Participants responses to factors which had a negative or positive impact on their physical activity indicated a number of significant differences between the groups (Table 4). The Decreased PA group showed significantly higher proportion of participants who identified Fear of COVID-19 (51%) and reduced motivation (71%) as factors which had a negative impact on the physical activity by comparison to the two other groups which reported these items much less frequently, *p* < 0.001 for both. The same Decreased PA group also more frequently reported having no time (33%) as a negative factor compared to the two other groups, *p* = 0.009. No significant differences were identified in reporting the closure of gyms or cancellation of sports events as negative factors impacting physical activity between groups. At least 80% of both the Increased PA and No change in PA groups reported having more time as a positive impact on their physical activity while only 31% of the Decreased PA group cited this as a positive impact factor on physical activity.

**Table 4:** Perceived positive and negative factors influencing levels of physical activity^#^.

**Factor**	**Total**	**No change in PA**	**Decreased PA**	**Increased PA**	***p*-value**
N	110	10 (9%)	70 (64%)	30 (27%)	
**Reasons for negative impact on PA**					
Fear of COVID-19	43 (39%)	3 (30%)	37 (51%)	3 (10%)	< 0.001
Closure of sports facility or gym	64 (58%)	6 (60%)	43 (61%)	15 (50%)	0.56
Cancellation of sport events	27 (25%)	1 (10%)	16 (23%)	10 (33%)	0.29
Reduced motivation	61 (55%)	5 (50%)	50 (71%)	6 (20%)	< 0.001
No time	26 (24%)	0 (0%)	23 (33%)	3 (10%)	0.009
Not impacted negatively	20 (18%)	3 (30%)	5 (7%)	12 (40%)	< 0.001
**Reasons for positive impact on PA**					
Supporting family members	14 (13%)	1 (10%)	8 (11%)	5 (17%)	0.77
More time available	55 (50%)	8 (80%)	22 (31%)	25 (83%)	< 0.001
More motivation	22 (20%)	1 (10%)	12 (17%)	9 (30%)	0.26
Not impacted positively	38 (35%)	1 (10%)	36 (53%)	1 (3%)	< 0.001

# Values in parentheses are column percentages. Significance values are from Chi-square test.

### Logistic regression analysis

Two different multivariate logistic regression analysis were completed to examine any demographic or physical activity behaviors which correlated with membership in the Increased PA and Decreased PA groups.

#### Factors associated with decreased physical activity during COVID-19

The results of multivariate logistic regression analysis model (Table 5) assessing possible demographic and physical activity behaviors pre-COVID-19 contribution to decreased PA during COVID-19 showed goodness of fit metrics indicating good calibration, with Pearson χ2(39) being 44, (*p* = 0.244), as well as good discrimination with an area under the curve (AUC) of 80% and Pseudo R2 was 20.9%. The adjusted odds ratios for odds of decrease in PA for different covariates are presented in Table 5. Females and those who reported negative impact of closed sports facility were observed to have lower odds of decrease in PA, although the effect was not significant. Those who reported fear of contracting COVID19 as the reason for negative impact on physical activity showed significantly higher odds of decrease in PA (OR=9.9, 95% CI: 3.27–30.37, *p* < 0.001). The odds of seeing a decrease in PA in those living alone were more than three times (OR=3.67, 95% CI: 1.25–10.76, *p* = 0.018) compared to those living at home with family. Similarly, those living with room-mates had almost 10 times the odds of experiencing a decrease in PA (OR=9.80, 95% CI: 1.95–49.32, *p* = 0.006) compared to those living at home with family.

**Table 5:** Factors associated with decreased PA (*N* = 110).

**Factor**	**Odds ratio**	**95% CI**	***p-*value**
Female	0.90	0.33–2.38	0.834
Physically active before COVID-19	0.77	0.29–2.06	0.610
Physical activity negatively impacted because of closed sports facility/gym	1.69	0.56–5.10	0.349
Physical activity negatively impacted because of fear of contracting Covid	9.9	3.27–30.37	< 0.001
Physical activity negatively impacted because of canceled sports events	0.35	0.10–1.17	0.088
**Living situation**			
At home with family	*Reference category*
Alone	3.67	1.25–10.76	0.018
With room-mates	9.80	1.95–49.32	0.006

#### Factors associated with increased physical activity during COVID-19

Multivariate logistic regression analysis model for possible demographic and physical activity behaviors pre-COVID-19 contribution to increased PA during COVID-19 (Table 6) also showed goodness of fit metrics indicating good calibration, with Pearson χ2(39) being 46.3, (*p* = 0.195), as well as good discrimination with an area under the curve (AUC) of 71.4% and Pseudo R2 was 10.9% indicating relatively weak predictive power for the model. Only one of the included covariates in the model had significant odds ratios predicting membership in the Increased PA group. Reporting use of a sports facility or gym pre-COVID-19 indicated participants were 80% less likely to experience an increase in PA during COVID-19, (OR = 0.21, 95% CI: 2.97–26.33, *p* = 0.011).

**Table 6:** Factors associated with increased PA (*N* = 110).

**Factor**	**Odds ratio**	**95% CI**	***p*-value**
Female	1.38	0.51–3.71	0.514
Physically active before COVID-19	2.20	0.82–5.90	0.117
Providing support to family	1.49	0.37–6.201	0.569
Use of smartwatch	0.58	0.22–1.57	0.291
Use of Sports facility / Gym before COVID-19 for physical activity	0.20	0.06–0.67	0.009
**Living situation**			
At home with family	*Reference category*
Alone	0.51	0.18–1.44	0.206
With room mates	0.53	0.09–3.00	0.481

## Discussion

The present study aimed to shed light on the potential negative consequences of the COVID-19 pandemic and lockdown on the physical activity of medical and nursing students. To our knowledge, this is the first study that investigates the pandemic's impact on physical activity and sedentary behavior of medical and nursing students within the MENA region. Our main findings indicate that 63% of the study's sample have experienced a decrease in their physical activity levels during the COVID-19 social distancing measures when compared to the 3 months prior. Further, 68% of our sample has reported increased sedentary behavior during the COVID-19 lockdown, with 79% of the Decreased PA group reporting increased sedentary behavior. In addition, we highlighted a significant difference between the groups' MET-minutes/week, where the Increased PA group had a median of 1605 MET-minutes/week compared to 424 MET-minutes/week and 1070 MET-minutes/week for the Decreased PA and No Change in PA groups, respectively.

In this current study we observed a similar trend to that reported by Constandt et al. (25) in that participants who exercised in a sports club or gym before the pandemic were less likely to experience an increase and more likely to experience a decrease in PA during the COVID-19 pandemic. 78% of this group of participants experienced a decline in physical activity, yet despite this observation, closure of gyms was deemed a statistically insignificant negative factor associated with decreased physical activity compared to no change or increased physical activity (*p* = 0.56). In contrast, fear of COVID-19 (< 0.001) was identified to be a statistically significant negative factor in decreased physical activity compared to No change in PA and Increased PA groups. This finding is in agreement with two other recent studies which show correlations between fear of COVID-19 and physical activity in German adolescents (35) and a Brazilian population sample (36). On the other hand, having more time available was cited as a primary reason for a positive impact on physical activity, as reported by at least 80% of both the Increased PA and No change in PA groups.

The completed regression analysis models also identified an interesting socio-demographic factor associated with changes in physical activity during the pandemic. In this study, students living away from home were up to 10 times more likely to experience decreased physical activity compared to those living at home with family. This is contrary to the findings presented by Romero-Blanco et al. (31) who reported that university students living away from home experienced a greater increase in physical activity levels during the pandemic compared to those living with family.

Current literature concerning the pandemic's impact on physical activity of medical and nursing students are limited and inconclusive. For instance, our results appear to be consistent with some past studies, where a significant reduction in physical activity was seen in the medical student body (37–39). Other studies report physical activity levels were not significantly impacted by the pandemic, suggesting that more available time and medical students being more likely to pursue healthy behaviors to be possible explanations (40, 41). However, it is important to note that prevalence of low physical activity was initially high in one of the aforementioned studies (40). As such, the lack of change, either positive or negative, should be considered critically.

Medical students are a population who are at risk for low physical activity and high sedentary behavior (42–45). In fact, university students, as a whole, are more likely to be less physically active than the general young adult population (12). As the importance of exercise and limiting sedentary behaviors cannot be understated (16, 46–48), universities should fully educate students on physical activity recommendations, allocate appropriate time for physical activity, maintain proper facilities, and inform students of home exercise regimes or other safe alternatives for the possible occurrence of another pandemic which requires similar social distancing measures (49, 50). Other avenues to increase physical activity is to encourage the practice of active behavior, including opting to use stairs, breaking sessions of prolonged sedentary behavior, walking or cycling to destinations, or participation in sports (51–53). Furthermore, it has been shown that teaching about physical activity and its associated benefits is limited throughout medical school. In a study by Williford et al. (54), 78% of participating physicians believe that there is a need for an exercise-focused course in medical school. Despite the majority of medical schools offering some type of physical activity education (55), the number of hours dedicated to the topic is deemed inadequate and leaves much to be desired. For instance, only 4.2 and 8.1 h in UK and US medical schools, respectively, are dedicated to physical activity education over the entirety of medical school (56, 57). Reasons for this include viewing other forms of medical education as more important, lack of dedicated staff or course time for such teaching, and a lack of student interest (55). To address this, focused education on physical activity should permeate the medical school curriculum, aiding medical students to recognize the importance of exercise and enabling them to recommend physical activity interventions in their future clinical practice (55).

While the present study highlights the negative impact of the COVID-19 lockdown on the physical activity of medical and nursing students, several limitations should be disclosed. To begin, the study is cross-sectional in nature, utilizing self-selection and convenience sampling in one institution in Bahrain. Despite the intended sample of 290 participants, we obtained 110 responses. Although we were able to find significant associations between variables and change in physical activity, a higher number of subjects could have made further insights possible, and a larger study is warranted to explore this subject further. Additionally, the results presented cannot be fully generalized to the entire medical or nursing student bodies in the MENA region. While the IPAQ questionnaire is validated, it remains a less reliable instrument than device-based tracking, leading to potential inaccuracies associated with self-reported physical activity and sedentary behavior. For physical activity levels prior to the COVID-19 lockdown, participants were fully reliant on their recollection, which may be prone to inaccuracy or overestimation. In addition, the IPAQ questionnaire does not differentiate between continuous and intermitted sedentary behavior; intermitted sedentary behavior has been associated with better health and metabolic outcomes (52).

## Conclusions

Compared to the young adult population, university students are at increased risk of experiencing decreases in physical activity levels during periods of social distancing measures during a pandemic. Steps should be taken by universities to educate students about physical activity and exercise in general. In the event of another pandemic, alternative forms of exercise should be promoted to all students, particularly those who are involved in team sports or use sports facilities and gyms. Information should also be made available that informs students about forms of exercise which present low risk of infection to address infection anxiety within this population. Additionally, appropriate support should be created to target students according to their specific living situations.

## Data availability statement

The raw data supporting the conclusions of this article will be made available by the authors, without undue reservation.

## Ethics statement

The studies involving human participants were reviewed and approved by Research Ethics Committee Royal College of Surgeons Medical University of Bahrain. The patients/participants provided their written informed consent to participate in this study.

## Author contributions

AA-H, DG, and NK contributed to the project conceptualization and methodology. AA-H and DG completed data collection. NK completed the analysis. DG managed project administration. All authors have contributed to edited, read, and agreed the submitted version of this manuscript.

## Funding

Open access fees have been provided by the School of Postgraduate Studies and Research, Royal College of Surgeons in Ireland Medical University of Bahrain.

## Conflict of interest

The authors declare that the research was conducted in the absence of any commercial or financial relationships that could be construed as a potential conflict of interest.

## Publisher's note

All claims expressed in this article are solely those of the authors and do not necessarily represent those of their affiliated organizations, or those of the publisher, the editors and the reviewers. Any product that may be evaluated in this article, or claim that may be made by its manufacturer, is not guaranteed or endorsed by the publisher.
